# Identifying hemodynamic factors associated with the rupture of anterior communicating artery aneurysms based on global modeling of blood flow in the cerebral artery network

**DOI:** 10.3389/fbioe.2024.1419519

**Published:** 2024-06-13

**Authors:** Yuqing Tian, Xiao Li, Jianjian Zhang, Bing Zhao, Fuyou Liang

**Affiliations:** ^1^ Department of Engineering Mechanics, School of Ocean and Civil Engineering, Shanghai Jiao Tong University, Shanghai, China; ^2^ Department of Radiology, Renji Hospital, Shanghai Jiao Tong University School of Medicine, Shanghai, China; ^3^ Department of Neurosurgery, Renji Hospital, Shanghai Jiao Tong University School of Medicine, Shanghai, China; ^4^ Key Laboratory of Hydrodynamics (MOE), School of Ocean and Civil Engineering, Shanghai Jiao Tong University, Shanghai, China; ^5^ State Key Laboratory of Ocean Engineering, School of Ocean and Civil Engineering, Shanghai Jiao Tong University, Shanghai, China; ^6^ World-Class Research Center “Digital Biodesign and Personalized Healthcare”, Sechenov First Moscow State Medical University, Moscow, Russia

**Keywords:** anterior communicating artery, aneurysm rupture, boundary conditions, hemodynamics, multivariate logistic regression

## Abstract

Anterior communicating artery (ACoA) aneurysms are more prone to rupture compared to aneurysms present in other cerebral arteries. We hypothesize that systemic blood flow in the cerebral artery network plays an important role in shaping intra-aneurysmal hemodynamic environment thereby affecting the rupture risk of ACoA aneurysms. The majority of existing numerical studies in this field employed local modeling methods where the physical boundaries of a model are confined to the aneurysm region, which, though having the benefit of reducing computational cost, may compromise the physiological fidelity of numerical results due to insufficient account of systemic cerebral arterial hemodynamics. In the present study, we firstly carried out numerical experiments to address the difference between the outcomes of local and global modeling methods, demonstrating that local modeling confined to the aneurysm region results in inaccurate predictions of hemodynamic parameters compared with global modeling of the ACoA aneurysm as part of the cerebral artery network. Motivated by this finding, we built global hemodynamic models for 40 ACoA aneurysms (including 20 ruptured and 20 unruptured ones) based on medical image data. Statistical analysis of the computed hemodynamic data revealed that maximum wall shear stress (WSS), minimum WSS divergence, and maximum WSS gradient differed significantly between the ruptured and unruptured ACoA aneurysms. Optimal threshold values of high/low WSS metrics were determined through a series of statistical tests. In the meantime, some morphological parameters of aneurysms, such as large nonsphericity index, aspect ratio, and bottleneck factor, were found to be associated closely with aneurysm rupture. Furthermore, multivariate logistic regression analyses were performed to derive models combining hemodynamic and morphological parameters for discriminating the rupture status of aneurysms. The capability of the models in rupture status discrimination was high, with the area under the receiver operating characteristic curve reaching up to 0.9. The findings of the study suggest that global modeling of the cerebral artery network is essential for reliable quantification of hemodynamics in ACoA aneurysms, disturbed WSS and irregular aneurysm morphology are associated closely with aneurysm rupture, and multivariate models integrating hemodynamic and morphological parameters have high potential for assessing the rupture risk of ACoA aneurysms.

## 1 Introduction

Intracranial aneurysms (IAs), featured by abnormal local enlargement of cerebral arteries, are a common vascular disease affecting approximately 1%–5% of the population (Brisman et al., 2006; Starke et al., 2013). Rupture of an IA usually causes subarachnoid hemorrhage, leading to a mortality rate of up to 50% (Schröder et al., 1997; Van Gijn et al., 2007), with over 40% of survivors experiencing severe disabilities (Amenta et al., 2012). IAs can develop in multiple cerebral arteries, with about 15.5% located at the anterior communicating artery (ACoA) (Morita et al., 2012). Blood flow patterns in the ACoA are highly complex, because multiple arteries converge and diverge here, which renders the blood flow prone to disturbance and sensitive to flow conditions in adjacent arteries. The intensity of flow disturbance in the ACoA may be augmented when an aneurysm that significantly alters local vascular morphology develops, which may further increase the risk of endothelial dysfunction and vascular damage (Ku et al., 1985; Malek et al., 1999). In fact, it has been found that ACoA aneurysms are particularly more prone to rupture compared to aneurysms located elsewhere in patients with multiple IAs (Lu et al., 2013). Another critical issue associated with the management of ACoA aneurysms is the relatively high risk of complications or fatal events subsequent to endovascular treatments compared with IAs located elsewhere (Park et al., 2018; Chen et al., 2020). Therefore, identifying ACoA aneurysms of high rupture risk has great clinical significance.

Previous studies have demonstrated the potential associations of many morphological and hemodynamic factors (e.g., larger aneurysm size, large aspect ratio, abnormally low/high/oscillatory wall shear stress) with aneurysm rupture (Chien et al., 2008; Backes et al., 2014; Li et al., 2018; Liang et al., 2018). However, it remains debatable whether such associations could be applied to all IAs. In particular, it was found that many ACoA aneurysms ruptured even though their sizes were small (Forget et al., 2001; Jeong et al., 2009; Joo et al., 2009), which implies that large aneurysm size and size-dependent hemodynamic disturbances might not be reliable factors for predicting the rupture risk of ACoA aneurysms. In this context, some studies attempted to explore factors associated with the formation and rupture of ACoA aneurysm from a more systemic viewpoint, e.g., considering the anatomical structure of the circle of Willis (CoW)) and associated hemodynamic characteristics. A study found that unilateral hypoplasia of the A1 segment of the anterior cerebral artery (ACA1) was detected in 68.1% of patients with ACoA aneurysms (Kwak et al., 1980). In comparison, such anatomical defect was found in only 10%–35% of adults free of cerebral vascular disease (Niederberger et al., 2010). Some other studies revealed that anatomical asymmetries of the CoW were associated with ACoA aneurysm instability and rupture (Marc et al., 2012; Tarulli et al., 2014; Stojanović et al., 2019). In general, anatomical variations in CoW would not cause brain ischemia, but will alter the distribution of blood flow within the CoW and blood flow patterns in specific cerebral arteries (Alastruey et al., 2007; Liang et al., 2011). Blood flow in the ACoA or an aneurysm there would be particularly sensitive to CoW variations given the specific location of the ACoA in the CoW, as has been demonstrated by previous studies (Karmonik et al., 2009; Hassan et al., 2011; Liang et al., 2016). Based on these previous findings, we hypothesize that systemically considering the anatomical variations in CoW and their hemodynamic effects would favor a more sophisticated exploration of hemodynamic factors related to the rupture of ACoA aneurysms.

Medical image-based computational fluid dynamics (CFD) techniques have been widely applied to address hemodynamic differences between ruptured vs unruptured IAs thereby identifying hemodynamic factors of potential value for predicting the risk of aneurysm rupture (Cebral et al., 2011; Xiang et al., 2011; Li et al., 2014). Most studies in this field built CFD models covering a limited physical region (usually confined to an IA and its adjacent arteries). The modeling strategy can effectively lower the burden of model reconstruction and reduce computational cost, thereby facilitating studies on large-scale IAs. The advantages however may be compromised by the reduced fidelity of numerical results due to the missing information on systemic hemodynamics that is critical for determining the inflow and outflow conditions of IAs. At this point, some studies have proved the importance of including sufficient upstream cerebral arteries for accurate computation of intra-aneurysmal hemodynamics (Castro et al., 2006; Pereira et al., 2013; Hodis et al., 2015; Hua et al., 2015; Xin et al., 2022). A recent study on a small number of ACoA aneurysms demonstrated that the computed time-averaged wall shear stresses in ACoA aneurysms by unilateral model and complete model of the CoW could differ up to 62% in certain cases (Yang et al., 2024).

In light of the aforementioned issues, we firstly conducted numerical experiments to investigate to what extend the selection of physiological boundaries in the modeling of ACoA aneurysm would affect the computed intra-aneurysmal hemodynamic quantities (herein simplified models confined to the local aneurysm region vs global models of the entire CoW). As we will show later, the two modeling strategies resulted in differential numerical results. Subsequently, we applied the global modeling strategy to forty ACoA aneurysms (containing twenty ruptured and twenty unruptured ones), yielding a series of numerical data for evaluating the hemodynamic differences between ruptured and unruptured aneurysms. In addition to the improvement in modeling method, we detailed the analysis of hemodynamic data by expanding wall shear stress (WSS) metrics for comparison and refining the determinant of threshold values. Finally, we combined hemodynamic and morphological parameters to establish mathematical models for predicting the rupture risk of ACoA aneurysms.

## 2 Materials and methods

### 2.1 Patients and clinical data

Computed tomography angiography (CTA) data were retrospectively collected from patients diagnosed with ACoA aneurysms at Renji Hospital (Shanghai, China). The use of the data for research has been approved by the hospital’s ethics committee. In consideration of global modeling strategy adopted in our study, only the aneurysms with high-quality medical images supporting the reconstruction of all major cerebral arteries were included. As a consequence, forty ACoA aneurysms were involved in our study, containing twenty ruptured ones and twenty unruptured ones. Preliminary image analysis showed that hypoplastic (underdeveloped) ACA1 was present in seven patients in the ruptured aneurysm group and in six patients in the unruptured aneurysm group, which implies that hypoplasia of the ACA1 may not be a factor related to the rupture risk of ACoA aneurysm, at least in the cohort of patients involved in the present study.

### 2.2 Image-based geometric model reconstruction and mesh generation

The geometric model of each ACoA aneurysm was reconstructed along with large cerebral arteries from CTA images using Mimics 16.0 (Materialise, Belgium). To control the complexity of model while maintaining the natural anatomical structure of the CoW, the following model reconstruction strategies were adopted: 1) all the large cerebral arteries constituting the CoW are maintained [i.e., A1 segment of left/right anterior cerebral artery (ACA1), anterior communicating artery (ACoA), P1 segment of left/right posterior cerebral artery (PCA1), and left/right posterior communicating artery (PCoA)]; 2) major afferent cerebral arteries [i.e., left/right internal carotid artery (ICA), basilar artery (BA)] are fully reconstructed based on available image data; and 3) reconstruction of efferent cerebral arteries were limited to the six largest ones [i.e., A2 segment of left/right anterior cerebral artery (ACA2), left/right middle cerebral artery (MCA), and P2 segment of left/right posterior cerebral artery (PCA2)] that deliver blood to different brain tissue regions. The reconstructed model for each patient was further treated by adding extension tubes to the inlets of the afferent arteries and the outlets of the efferent arteries. The length of the extension tube connected to each inlet/outlet was set to be 15 times/6 times of the vascular diameter (Myers et al., 2001). The addition of the extension tubes was expected to minimize the influence of imposed pressure or flow velocity distribution at the inlets/outlets on the flow patterns in the aneurysm and CoW.

Mesh generation based on the reconstructed geometric model was implemented in Fluent Meshing (Fluent, ANSYS Inc., USA). Triangle surface meshes were firstly created on the vascular walls, followed by the division of the inner fluid domain with tetrahedral elements. Finally, ten layers of prism elements were mapped along the vascular wall to replace the tetrahedral elements in the near-wall regions. The thickness of the first prism layer adjacent to the wall was set to be 0.005 mm, which was increased by a ratio of 1.25 towards the inner layers. The prism element layers, given the regular mesh topology and smooth changes in mesh size from the wall towards the interior, were expected to improve the precision of flow simulation in the near-wall region where velocity gradient is large (Xu et al., 2018a). Mesh independence analysis was performed on a randomly selected model, by varying the maximum size of the tetrahedral elements from 0.16 mm to 0.14 mm and 0.12 mm in sequence. It is noted that the minimum-to-maximum mesh size ratio was fixed at 1/16. The results showed that the relative error of the computed time-averaged WSS (TAWSS) at the entire aneurysm wall decreased from 1.747% to 0.555% following the reduction in mesh size. Therefore, the maximum size of the tetrahedral elements was set at 0.14 mm and was applied to all the models. The resulting mesh models contained 8 million to 25 million elements, depending on the size and complexity of the geometric models.

### 2.3 Setup of computational model

**FIGURE 1:**
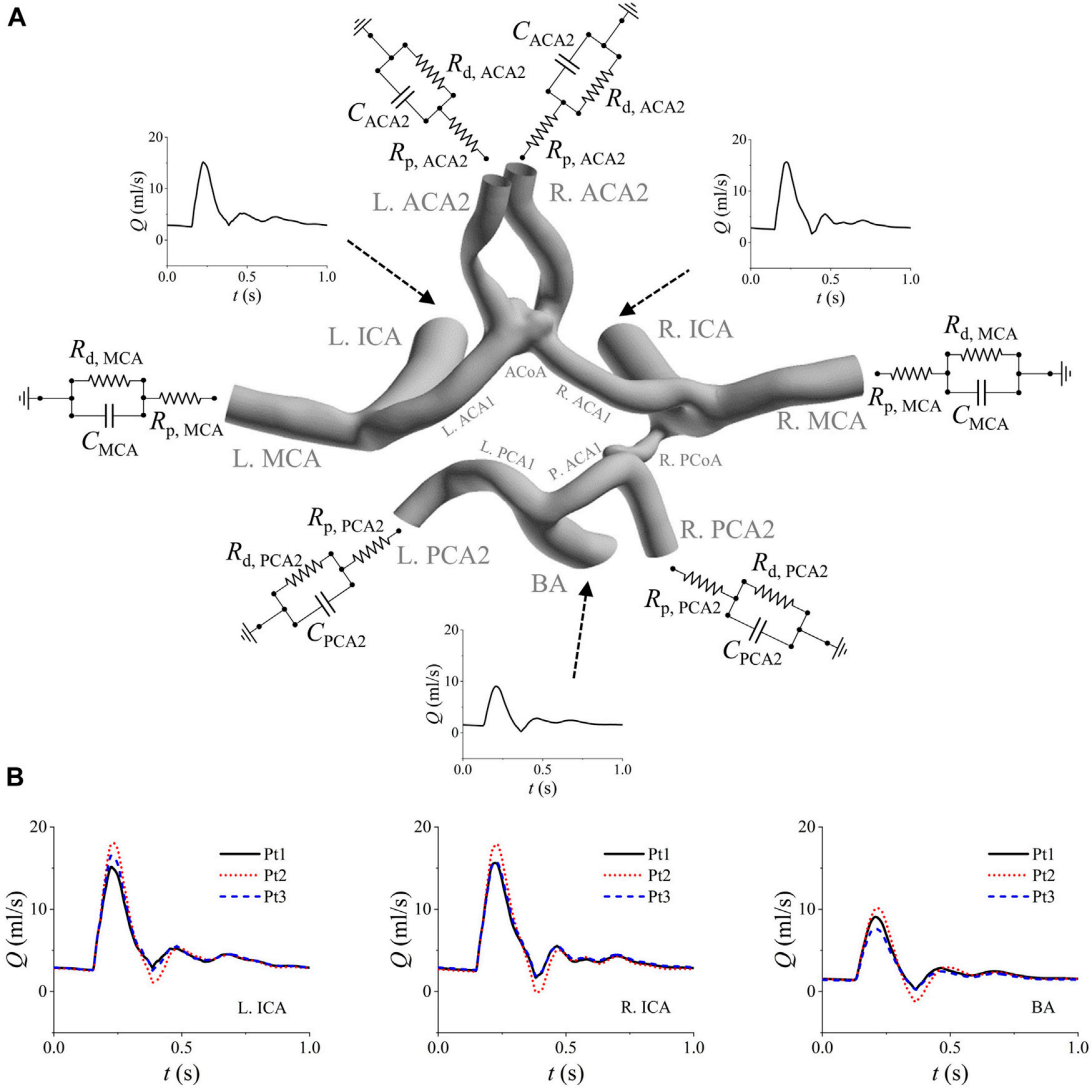
Boundary conditions assigned to the model of a patient (Pt1) **(A)**, and the comparison of the simulated inflow waveforms for three patients **(B)**. The inflow waveforms are those in the left ICA, right ICA, and BA. The RCR models are connected to the outlets of the six efferent arteries (i.e., left/right MCA, left/right ACA2, and left/right PCA2). Note that the added extension tubes are not shown in panel **(A)**.

Flow conditions imposed to the boundaries of a model have important influence on the outcome of numerical simulation (Xu et al., 2018b; Steinman and Pereira, 2019; Mehdi et al., 2021). However, blood flow velocities/rates in cerebral arteries are not routinely measured in clinical diagnosis or treatment of IAs (Xin et al., 2022). In this study, we employed a zero-one dimensional (0-1D) model of the cardiovascular system (Liang et al., 2011) to simulate the flow waveforms in cerebral arteries. The model was partly personalized by incorporating the detailed structural and geometrical information of the cerebral artery network (extracted from the 3D geometric model reconstructed based on CTA images), as such it could account for the influence of patient-specific cerebral artery anatomy while simulating flow waveforms in cerebral arteries. The simulated flow waveforms in the afferent cerebral arteries were then used to prescribe the inlet flow conditions of each 3D model. Figure 1A shows the model reconstructed for a patient with prescribed boundary conditions. The inflow waveforms simulated (with 0-1D models) for three patients are compared in Figure 1B. Considerable inter-patient differences in the magnitudes of the flow waveforms in the three afferent arteries were observed, reflecting the influence of patient-specific geometrical size and structure of the cerebral artery network. The simulated flow waveforms were similar in shape with *in vivo* measurements reported in previous studies (Reymond et al., 2009; Zhou et al., 2020). The outlets of the efferent cerebral arteries were each connected to a three-element Windkessel model (i.e., RCR model) which plays a role of controlling the flow division among the efferent cerebral arteries. The RCR model was formulated in form of Eq. 1, where *Q* and *P* represent the volume flow rate and pressure at the outlet, which are related via two resistances (*R*_p_, *R*_d_) and a compliance (*C*) (Alastruey et al., 2008; Pahlevan et al., 2011). The total resistance [*R*_T_ = *R*_p_ + *R*_d_, with *R*_p_/*R*_T_ = 0.2 (Stergiopulos et al., 1992)] in each outlet model was tuned so that the computed flow division among the efferent cerebral arteries is consistent with the population-averaged physiological data (Liang et al., 2011). All vascular walls in the 3D model were assumed to be rigid to which the no-slip boundary condition was imposed.

$$\frac{dP}{dt}=R_{p}\frac{dQ}{dt}+\frac{1}{R_{d}C}\left(\left(R_{p}+R_{d}\right)Q-P\right)$$ (1)

Blood flows in cerebral arteries and aneurysms were modeled as non-Newtonian incompressible fluid and laminar flow regime was assumed. Accordingly, blood flows were governed by the continuity equation (Eq. 2) and momentum conservation equation (Eq. 3).

∇⋅u=0 (2)

$$\frac{\partial u}{\partial t}+u\cdot \nabla u=-\frac{1}{\rho }\nabla p+\nabla \cdot \left(\frac{\mu }{\rho }\nabla u\right)$$ (3)

$$\mu =\mu_{\infty }+\left(\mu_{0}-\mu_{\infty }\right){\left[1+{\left(\lambda \dot{\gamma }\right)}^{2}\right]}^{\left(n-1\right)/2}$$ (4)

where *t* is the time, ***u*** is the flow velocity vector, *p* is the pressure, and *ρ* (1,060 kg/m^3^) is the blood density. The non-Newtonian rheological properties of blood were represented by modeling the dynamic viscosity as a function of shear rate (Eq. 4, the Carreau model), where *μ*_0_ (0.056 Pa·s) and *μ*_∞_ (0.00345 Pa·s) stand for the dynamic viscosities of blood when the shear rate approaches zero and becomes infinitely large, respectively, *λ* and *n* are constants, with their values being set at 3.313 s and 0.3568, respectively (Johnston et al., 2004; Nagargoje et al., 2021).

The governing equations of blood flow were numerically solved along with the boundary conditions with the finite volume method using a CFD package (Fluent, ANSYS Inc., USA). Second-order schemes were adopted for both the temporal and spatial terms. The outlet models were solved in conjunction with the 3D model via user-defined functions (UDFs) embedded in Fluent. Specifically, the pressures at the 3D model outlets were updated via solving Eq. 1 (with the explicit Euler method) during internal iterations within each numerical time step. The numerical time step was fixed at 0.001s and the threshold of residuals for judging convergence at every time step was set to be 1E-4. Numerical simulations were conducted under pulsatile flow conditions. Each set of numerical simulation was run continually for two cardiac cycles to achieve periodic solutions.

### 2.4 Data analysis

Analysis of hemodynamic quantities was focused on WSS at aneurysm wall. Under pulsatile flow conditions, WSS vector ( τ ) changes in magnitude and direction. To facilitate quantitative analysis, various WSS metrics have been defined in the literature. Typical WSS metrics include the time-averaged WSS (TAWSS, averaged magnitude of ***τ*** over a cardiac cycle) and oscillatory shear index (OSI, a measure of directional changes in WSS over a cardiac cycle) (Xin et al., 2022). In the study, we further considered some metrics characterizing the spatial variations of WSS, since WSS topology has been demonstrated to affect the initiation and progression of vascular disease. For instance, negative WSS divergence, which is often detected in wall regions where the wall shear stress vectors converge toward a focal point, can predict the localization of atheroprone low-density lipoprotein (LDL) deposition (Mazzi et al., 2022; Tian et al., 2023), whereas high WSS gradient and gradient oscillation are associated with endothelium injury and aneurysm formation (Shimogonya et al., 2009; Metaxa et al., 2010). Herein, three spatial WSS metrics were considered, namely, WSS divergence (WSSD, Eq. 5), WSS gradient (WSSG, Eq. 6), and gradient oscillatory number (GON, Eq. 7), which were firstly calculated at each time step and then integrated over a cardiac cycle.

$$\text{WSSD}=\int_{0}^{T}\frac{\nabla \cdot \tau }{T}dt$$ (5)

$$\text{WSSG}=\int_{0}^{T}\frac{\left|\left.\left.\nabla \right|\tau \right|\right|}{T}dt$$ (6)

$$\text{GON}=1-\left|\int_{0}^{T}\left.\left.\nabla \right|\tau \right|dt\right|/\int_{0}^{T}\left|\left.\left.\nabla \right|\tau \right|\right|dt$$ (7)

To further facilitate the comparison of WSS metrics between ruptured and unruptured aneurysms, the maximum, minimum and mean values of each WSS metric over the aneurysm wall were derived, which are denoted, for example, as maxTAWSS, minTAWSS, and SA-TAWSS for TAWSS. Furthermore, we calculated the total area of aneurysm walls exposed to WSS metrics higher or lower than certain thresholds. For TAWSS, AHS and ALS represent the areas of local aneurysm walls exposed to TAWSS > WSS_HT_ or TAWSS < WSS_LT_, where WSS_HT_ and WSS_LT_ are the thresholds of high TAWSS and low TAWSS, respectively. Similar area indices were defined for high OSI (AHI, OSI > OSI_HT_), low WSSD (ALD, WSSD < WSSD_LT_), high WSSG (AHG, WSSG > WSSG_HT_) and high GON (AHN, GON > GON_HT_), respectively. Each area index was further divided by the total area of the aneurysm wall to derive the relative area index (i.e., area ratio). It is noted that the thresholds of each WSS metric were not derived directly from the literature or estimated empirically, but determined via a series of statistical tests so that the area indices differ most evidently between ruptured and unruptured aneurysms.

In addition to WSS metrics, some morphological parameters of aneurysm were considered, which included aneurysm dome area (DA), volume (V_aneu_), nonsphericity index [NSI, NSI = 1-(18π)^1/3^V_aneu_^2/3^/A], height (H, the longest perpendicular distance to the neck plane), width (W, the largest diameter perpendicular to H), diameter of the aneurysm neck (D_neck_), height-to-width ratio (HWR, HWR = H/W), aspect ratio (AR, AR = H/D_neck_), and bottleneck factor (BF, BF = W/D_neck_) (Ma et al., 2004; Chien et al., 2018; Xin et al., 2022).

The Mann-Whitney *U* test was performed to compare parameters between ruptured and unruptured aneurysms. All the tests were two-tailed and a *p* value of <0.05 was used to judge statistical significance. The Spearman’s rank test was conducted to evaluate the correlation between any two parameters. Multivariate logistic regression analyses were employed to derive models for discriminating the rupture status of aneurysms, with the performances of the models being evaluated by receiver operating characteristics (ROC) analysis. All the statistical analyses were implemented in SPSS 26 (IBM, Armonk, NY).

### 2.5 Influence on numerical results of physical boundary truncation strategies in image-based model reconstruction

We randomly selected two patients (Pt4 with a ruptured ACoA aneurysm and Pt5 with an unruptured ACoA aneurysm) for test. For each patient, two geometric models were reconstructed based on medical images with different physical boundary truncation strategies: one is a global model containing the ACoA aneurysm and all the major cerebral arteries, and the other one is a local model containing the ACoA aneurysm and the surrounding arteries (i.e., left and/or right ACA1, and left/right ACA2). The outlets of the two models were supported by the same RCR models, while the flow waveforms imposed at the inlets of the local model were derived from the numerical results of the global model. The boundary condition settings guaranteed that the global and local models had the same flow rates through the ACA1s and flow division between the left and right ACA2s.

**FIGURE 2:**
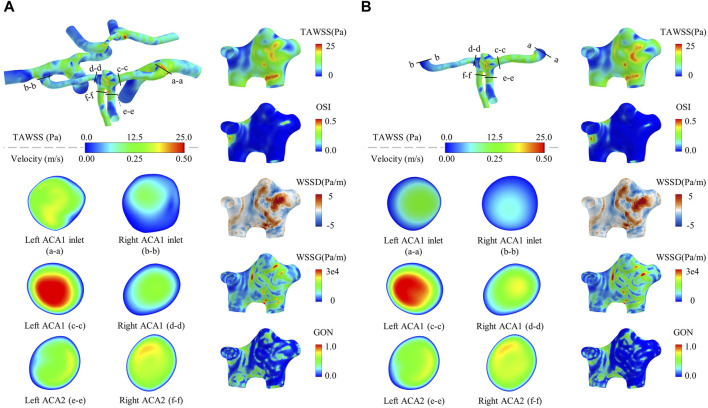
Contour maps of WSS metrics in the aneurysm region and flow velocities in six representative vascular cross sections computed by a global model **(A)** compared with those computed by a local model **(B)**. The models are constructed for a randomly selected patient (Pt4). The flow velocity contours are drawn based on the time-averaged flow velocities over a cardiac cycle. It is noted that the cross-sectional shapes of the ACA1 inlets differ slightly between the local and global models because the ACA1 inlet surfaces were slightly smoothened to facilitate the connection to the extension tubes in the case of local modeling.

Figure 2 shows the contour maps of WSS metrics in the aneurysm region and flow velocities in six representative vascular cross sections computed by the global and local models of a patient (Pt4). Visually, all the WSS metrics differed between the two models in terms of magnitude and spatial distribution. As for the flow velocity contours, evident between-model differences were observed for those in the cross sections of the left ACA1 and right ACA1, especially at the ACA1inlets (i.e., cross sections ‘a-a’ and ‘b-b’), whereas the differences were relatively small in the cross sections of the left and right ACA2s. Mechanisms underlying the observations are related to the role of upstream arteries in shaping the 3D patterns of flows entering the ACA1s. In comparison with the global model, the local model, though imposed with the same volume flow rates in the ACA1s, lost the 3D flow information in the upstream arteries that would pose influence on flow patterns in the ACA1s, which ultimately resulted in differential predictions of WSS metrics in the ACoA aneurysm. To enhance quantitative comparison between the outcomes of the two types of models, the space-averaged values of some WSS metrics over the ACoA aneurysm wall were calculated and provided in Table 1. The between-model differences were within ±10% for SA-TAWSS and SA-WSSG, but were much larger for SA-WSSD. In addition, the between-model differences differed significantly between the two patients. These results indicate that errors in numerical results associated with local modeling are somehow uncertain and are dependent on patient-specific anatomical features of the cerebral artery network and ACoA aneurysm. In this sense, global modeling may be a reliable approach to minimizing the influence of improper physical boundary truncation, thereby improving the physiological fidelity of numerical results, although the computational cost will increase significantly compared with local modeling. The modeling strategy was adopted for all the ACoA aneurysms involved in the study.

**TABLE 1:** Comparison of WSS metrics computed by the global and local models.

Patient	Model type	SA-TAWSS (Pa)	Difference (%)	SA-WSSD (Pa/m)	Difference (%)	SA-WSSG (Pa/m)	Difference (%)
Pt4 (unruptured)	Global	7.694	—	−0.0278	—	8,237	—
	Local	8.045	4.56	−0.0576	107.15	8,555	3.86
Pt5 (ruptured)	Global	6.628	—	0.0657	—	7,857	—
	Local	6.260	−5.55	0.0784	19.41	7,328	−6.74

## 3 Results

### 3.1 Comparisons of morphological and hemodynamic parameters between ruptured and unruptured aneurysms

**FIGURE 3:**
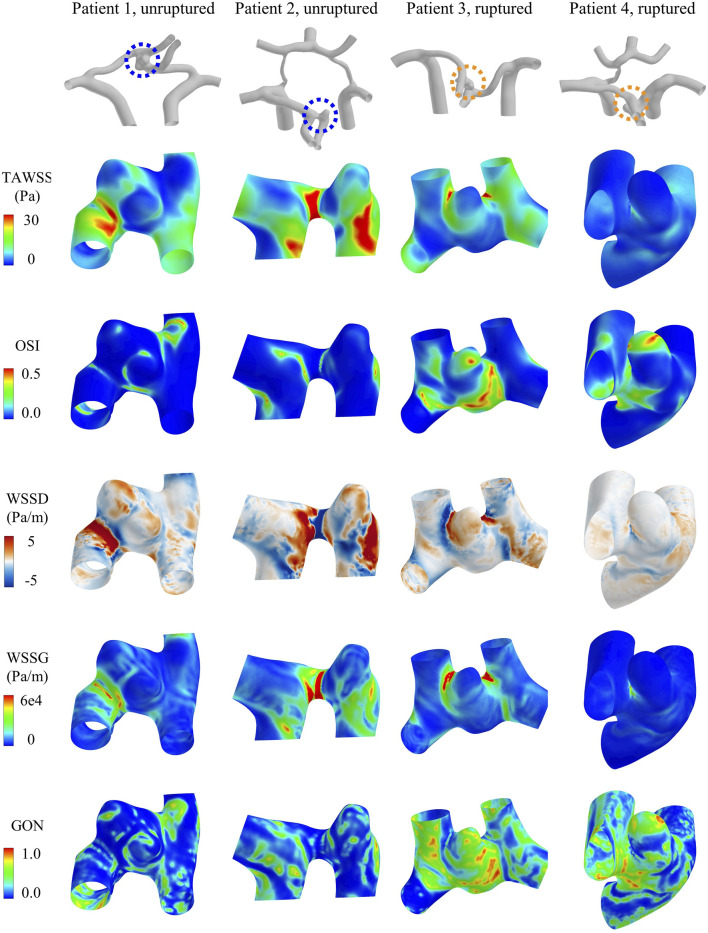
Contour maps of WSS metrics (i.e., TAWSS, OSI, WSSD, WSSG, and GON) in four representative aneurysms [two unruptured (left) vs two ruptured (right)].

Hemodynamic simulations were performed for all the forty ACoA aneurysms. Figure 3 shows the contour maps of model-predicted WSS metrics in four ACoA aneurysms (two unruptured vs two ruptured). The spatial distributions of the WSS metrics were highly complex, exhibiting aneurysm-specific characteristics. However, no specific characteristics were found to clearly differ between the unruptured and ruptured aneurysms.

Table 2 shows the statistical comparisons of the morphological and hemodynamic parameters between the twenty ruptured aneurysms and twenty unruptured aneurysms. The data are presented in form of mean ± standard deviation. Four out of nine morphological parameters were found to differ statistically between the ruptured and unruptured aneurysms, which included the nonsphericity index (NSI), height-to-width ratio (HWR), aspect ratio (AR), and bottleneck factor (BF). All the parameters were larger in the ruptured aneurysm group. Interestingly, the parameters measuring the absolute size of aneurysm, such as the dome area, volume, and aneurysm height, did not show statistically significant between-group differences. As for hemodynamic parameters, three WSS metrics (i.e., maxTAWSS, maxWSSG and minWSSD) were found to differ significantly between the unruptured and ruptured aneurysms. Specifically, maxTAWSS and maxWSSG were larger while minWSSD was smaller in the ruptured aneurysm group. The ruptured aneurysm group had an overall lower minTAWSS, but the between-group differences did not reach statistical significance. No between-group difference was identified for any space-averaged (SA) WSS metrics.

**TABLE 2:** Statistical comparisons of morphological and hemodynamic parameters between ruptured aneurysms (*n* = 20) and unruptured aneurysms (*n* = 20).

Parameter	Ruptured (*n* = 20)	Unruptured (*n* = 20)	*p* value
DA (m^2^)	8.886E-5 ± 6.922E-5	6.041E-5 ± 5.036E-5	0.096
V_aneu_ (m^3^)	9.101E-8 ± 9.650E-8	6.179E-8 ± 6.838E-8	0.165
NSI	2.955E-1 ± 3.750E-2	2.666E-1 ± 2.364E-2	0.004**
H(m)	4.426E-2 ± 2.214E-2	3.172E-2 ± 1.587E-2	0.060
W(m)	6.246E-2 ± 2.247E-2	5.384E-2 ± 1.737E-2	0.242
D_neck_(m)	5.618E-2 ± 1.156E-2	5.770E-2 ± 1.319E-2	0.620
HWR	7.094E-1 ± 2.414E-1	5.724E-1 ± 1.552E-1	0.046*
AR	7.628E-1 ± 2.673E-1	5.298E-1 ± 1.772E-1	0.004**
BF	1.098E+0 ± 2.689E-1	9.177E-1 ± 1.126E-1	0.049*
maxTAWSS(Pa)	59.980 ± 30.297	38.563 ± 26.443	0.009**
minTAWSS(Pa)	0.762 ± 0.357	1.234 ± 1.017	0.081
SA-TAWSS(Pa)	7.807 ± 3.063	8.732 ± 6.180	0.799
maxOSI	0.468 ± 0.034	0.465 ± 0.029	0.478
SA-OSI	0.060 ± 0.039	0.064 ± 0.037	0.602
maxWSSG(Pa/m)	2.838E5 ± 3.184E5	2.206E5 ± 5.963E5	0.001**
SA-WSSG(Pa/m)	1.115E4 ± 5.986E3	1.101E4 ± 7.822E3	0.659
minWSSD (Pa/m)	−15.276 ± 8.323	−9.224 ± 9.552	0.003**
SA-WSSD (Pa/m)	0.170 ± 0.241	0.226 ± 0.245	0.602
maxGON	0.880 ± 0.099	0.876 ± 0.059	0.414
SA-GON	0.228 ± 0.076	0.249 ± 0.077	0.289

*represents 0.01 < *p* < 0.05 and **represents *p* < 0.01.

**FIGURE 4:**
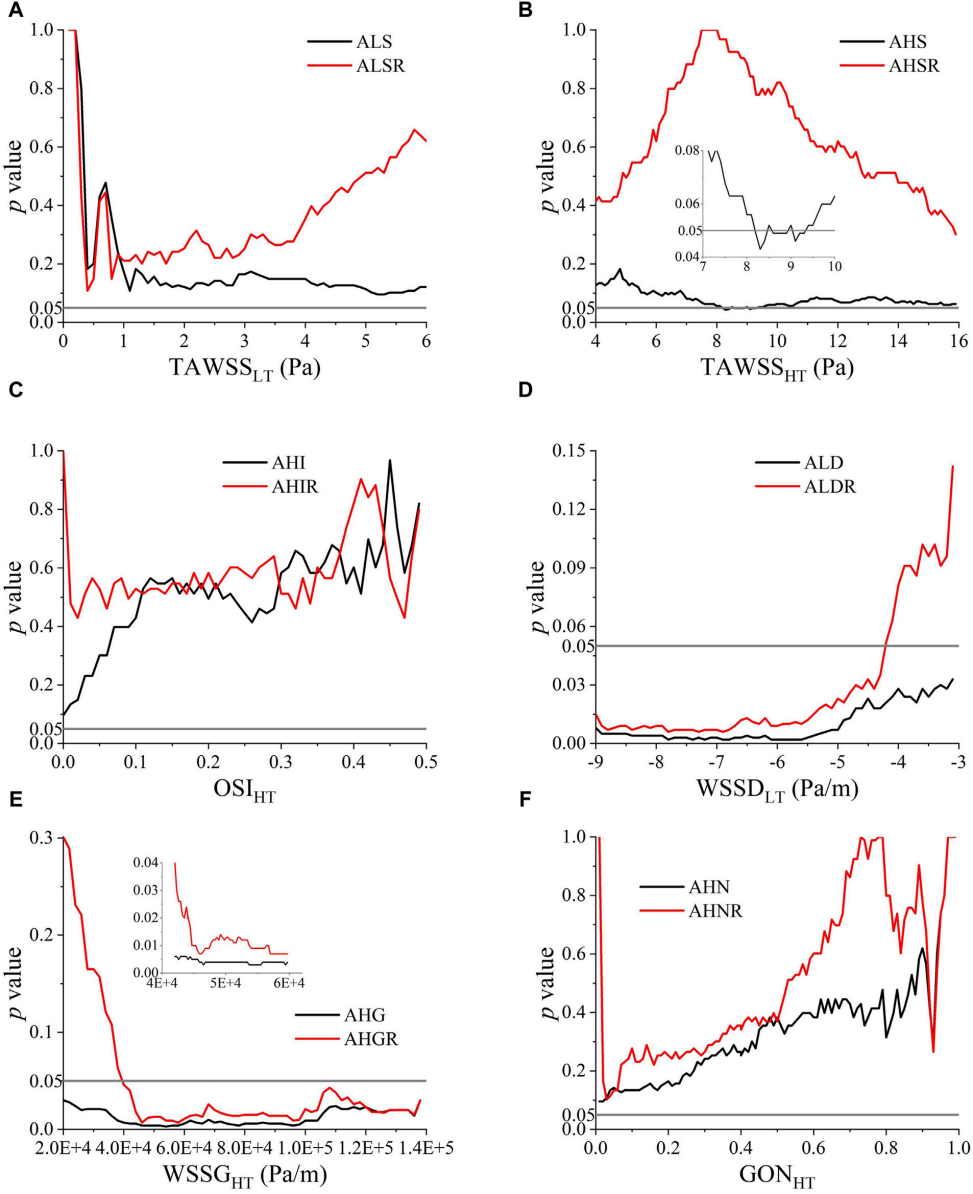
*p* values of Mann-Whitney *U* test for comparing the ruptured and unruptured aneurysms in terms of the area and area ratio of aneurysm walls exposed to high/low WSS metrics determined by varying high threshold (HT) or low threshold (LT): **(A)** TAWSS_LT_, **(B)** TAWSS_HT_, **(C)** OSI_HT_, **(D)** WSSD_LT_, **(E)** WSSG_HT_, **(F)** GON_HT_.

Although the space-averaged WSS metrics did not exhibit significant between-group differences, there still exists the possibility that WSS metrics limited to certain ranges differ between groups and could be used to discern the rupture status of ACOA aneurysms. Therefore, we further performed statistical analyses to explore the high or low thresholds of WSS metrics (herein, TAWSS, OSI, WSSD, WSSG, and GON). To facilitate quantitative analysis, the area and area ratio (relative to the wall area of aneurysm) of walls exposed to WSS metric values lower or higher than a given threshold (low threshold (LT), or high threshold (HT)) were calculated, which were subsequently compared between the ruptured and unruptured aneurysms by means of Mann-Whitney *U* test. To ensure that the thresholds are determined in a robust way, the candidate threshold of each WSS metric was varied in a large range, with the *p* value outputted from the Mann-Whitney *U* test being used as the criterion for judging the performance of the threshold. The *p* values are plotted against the varying thresholds in Figure 4. A valley was discernible on the *p* value–threshold curves of most WSS metrics, indicating the existence of an optimal threshold at which the *p* value reaches a local minimum. Notably, WSSD and WSSG exhibited a broad range of thresholds at which the corresponding *p* values are below 0.05, which implies that the power of ALD (area of low WSSD) and AHG (area of high WSSG) in discriminating the rupture status of ACoA aneurysms is less sensitive to the choice of threshold. In contrast, no thresholds resulting in a *p* value lower than 0.05 were found for low TAWSS, high OSI, and high GON. As for high TAWSS, the *p* value decreased to <0.05 when the threshold value was in the range of 8–10 Pa, but again increased over 0.05 following further increase of the threshold value. Based on the results, five area indices, namely, AHS (area of high TAWSS), ALD, AHG, ALDR (ALD ratio), and AHGR (AHG ratio), were found to differ significantly between the ruptured and unruptured aneurysms. The thresholds of the corresponding WSS metrics (i.e., TAWSS, WSSD, WSSG) were set at TAWSS_HT_ = 8.3 Pa, WSSD_LT_ = −6 Pa/m, and WSSG_HT_ = 55,000 Pa/m, yielding *p* values of 0.043, 0.002, 0.003, 0.008, and 0.009, respectively.

### 3.2 Performances of individual morphological and hemodynamic parameters for discriminating the rupture status of aneurysm

Receiver operating characteristic (ROC) analysis was carried out to evaluate the capability of each individual morphological/hemodynamic parameter in discriminating the rupture status of aneurysms. Table 3 summarizes the results of ROC analyses for all the parameters. maxWSSG exhibited the highest capacity of discernment, achieving an area under the ROC curve (AUC) of 0.7925, followed by ALD, minWSSD, AHG, NSI, and AR whose AUCs were all above 0.75. If the sensitivity-specificity balance was further considered, maxWSSG, minWSSD, AHG, and NSI mildly outperformed ALD and AR.

**TABLE 3:** Results of ROC analyses for individual morphological and hemodynamic parameters regarding their performances in discriminating the rupture status of aneurysms. The data were presented in form of AUC and sensitivity/specificity at an optimal threshold of each parameter.

Parameter	AUC	Threshold	Sensitivity	Specificity
NSI	0.7650	0.276	0.70	0.85
HWR	0.6850	0.578	0.75	0.65
AR	0.7625	0.642	0.70	0.80
BF	0.6825	1.117	0.40	0.95
maxTAWSS	0.7375	35.381	0.85	0.65
minWSSD	0.7725	−10.125	0.75	0.80
maxWSSG	0.7925	9.756E05	0.80	0.75
AHS	0.6850	8.820E-06	0.90	0.55
ALD	0.7775	1.260E-07	0.80	0.70
AHG	0.7675	2.845E-07	0.80	0.75
ALDR	0.7425	0.098	0.85	0.65
AHGR	0.7375	0.175	0.85	0.65

### 3.3 Derivation of multivariate models for predicting the rupture status of aneurysm

We further explored whether combining different parameters could enhance the discrimination of aneurysm rupture status beyond individual parameters by means of multivariate logistic regression analysis. In order to minimize the influence of multicollinearity caused by variable intercorrelations on the outcome of regression analysis, Spearman’s rank correlation analysis was firstly carried out to identify independent variables. The results are summarized in Table 4, where it was observed that maxWSSG and AHG were closely intercorrelated, and were correlated strongly with many other hemodynamic parameters, such as maxTAWSS, minWSSD, ALD, and ALDR. The two key morphological parameters (i.e., NSI and AR, which were identified by single-variate ROC analysis to be associated with aneurysm rupture) were independent, and were uncorrelated or correlated weakly with maxWSSG and AHG. Based on the above results, we selected two sets of parameters (one set consisting of maxWSSG, NSI and AR, the other set consisting of AHG, NSI and AR) as the independent variables in the multivariate logistic regression analysis. Given the twenty aneurysm rupture events in the dataset, the number of events per variable (EPV) (20 divided by 3) was about 7, which surpasses the lower limit of EPV (≥5) in logistic regression analysis for binary problem (herein, discriminating the ruptured status of aneurysm) (Peduzzi et al., 1996; Vittinghoff and McCulloch, 2007). To reduce potential roundoff errors caused by the differential magnitudes of parameters in multivariate regression analysis, each parameter was normalized by its mean value in all the aneurysms (herein the normalized parameter is denoted by a ‘*’ subscript). In addition, since parameter values might be nonlinearly related to the rupture status of aneurysm, single-variate logistic regression analysis was conducted for each parameter to determine the order of polynomial regression model with the best predictive performance (evaluated with ROC analysis). The results indicated that the optimal orders of single-variate polynomial models were second, fifth, fourth, and fifth for maxWSSG, AHG, NSI, and AR, respectively. However, when high-order polynomials of all the parameters are included in a multivariate model, the degrees of freedom (i.e., the number of predictors) will increase significantly, leading to low sample size corresponding to each individual predictor thereby augmenting the risk of overfitting (Babyak, 2004). With this in mind, we set the highest order of polynomials at second for maxWSSG and AHG, whereas first for NSI and AR in multivariate logistic regression analyses based on the trends of changes in AUC with the polynomial order in the single-variate logistic regression analyses so that the sample size was 10 [40 (number of aneurysms) divided by 4 (number of predictors)], fulfilling the lower limit of sample size (>8) in regression analysis as recommended by a recent study (Jenkins and Quintana-Ascencio, 2020). The multivariate logistic regression analyses with the two sets of parameters yielded two models predicting the probability of aneurysm rupture.

**TABLE 4:** Spearman’s rank coefficients of the correlations between morphological/hemodynamic parameters of aneurysms.

Paramaetr	NSI	HWR	AR	BF	maxTAWSS	minWSSD	maxWSSG	AHS	ALD	AHG	ALDR	AHGR
NSI	1.000	−0.116	0.188	0.436**	0.317*	−0.333*	0.321*	0.222	0.367*	0.352*	0.275	0.249
HWR	−0.116	1.000	0.857**	0.047	0.053	−0.050	0.032	0.251	0.064	−0.002	−0.129	−0.171
AR	0.188	0.857**	1.000	0.488**	0.258	−0.257	0.211	0.494**	0.290	0.244	0.040	0.034
BF	0.436**	0.047	0.488**	1.000	0.465**	−0.462**	0.385*	0.621**	0.494**	0.465**	0.326*	0.326*
maxTAWSS	0.317*	0.053	0.258	0.465**	1.000	−0.896**	0.887**	0.666**	0.856**	0.915**	0.811**	0.874**
minWSSD	−0.333*	−0.050	−0.257	−0.462**	−0.896**	1.000	−0.900**	−0.676**	−0.914**	−0.848**	−0.891**	−0.825**
maxWSSG	0.321*	0.032	0.211	0.385*	0.887**	−0.900**	1.000	0.485**	0.800**	0.849**	0.791**	0.859**
AHS	0.222	0.251	0.494**	0.621**	0.666**	−0.676**	0.485**	1.000	0.758**	0.630**	0.628**	0.483**
ALD	0.367*	0.064	0.290	0.494**	0.856**	−0.914**	0.800**	0.758**	1.000	0.886**	0.931**	0.817**
AHG	0.352*	−0.002	0.244	0.465**	0.915**	−0.848**	0.849**	0.630**	0.886**	1.000	0.838**	0.933**
ALDR	0.275	−0.129	0.040	0.326*	0.811**	−0.891**	0.791**	0.628**	0.931**	0.838**	1.000	0.880**
AHGR	0.249	−0.171	0.034	0.326*	0.874**	−0.825**	0.859**	0.483**	0.817**	0.933**	0.880**	1.000

**indicates a *p* value < 0.01, and *indicates a *p* value in the range of 0.01 ≤ *p* < 0.05.

For maxWSSG, NSI and AR, the regression model was expressed as

$${\text{odd}}_{\text{rp},1}=\exp\;\left[-12.268+2.638{\text{maxWSSG}}^{*}-0.374{\left({\text{maxWSS}G}^{*}\right)}^{2}+8.131{\text{NSI}}^{*}+3.246{\text{AR}}^{*}\right]$$ (8)

Whereas for AHG, NSI and AR, the regression model was written as

$${\text{odd}}_{\text{rp},2}=\exp\;\left[-14.027+2.927{\text{AHG}}^{*}-0.854{\left({\text{AHG}}^{*}\right)}^{2}+10.076{\text{NSI}}^{*}+3.524{\text{AR}}^{*}\right]$$ (9)

By definition, the probability of aneurysm rupture was calculated as

$$p_{\text{rp}}=\frac{{\text{odd}}_{\text{rp},i}}{1+{\text{odd}}_{\text{rp},i}}\;\left(i=1,2\right)$$ (10)

**FIGURE 5:**
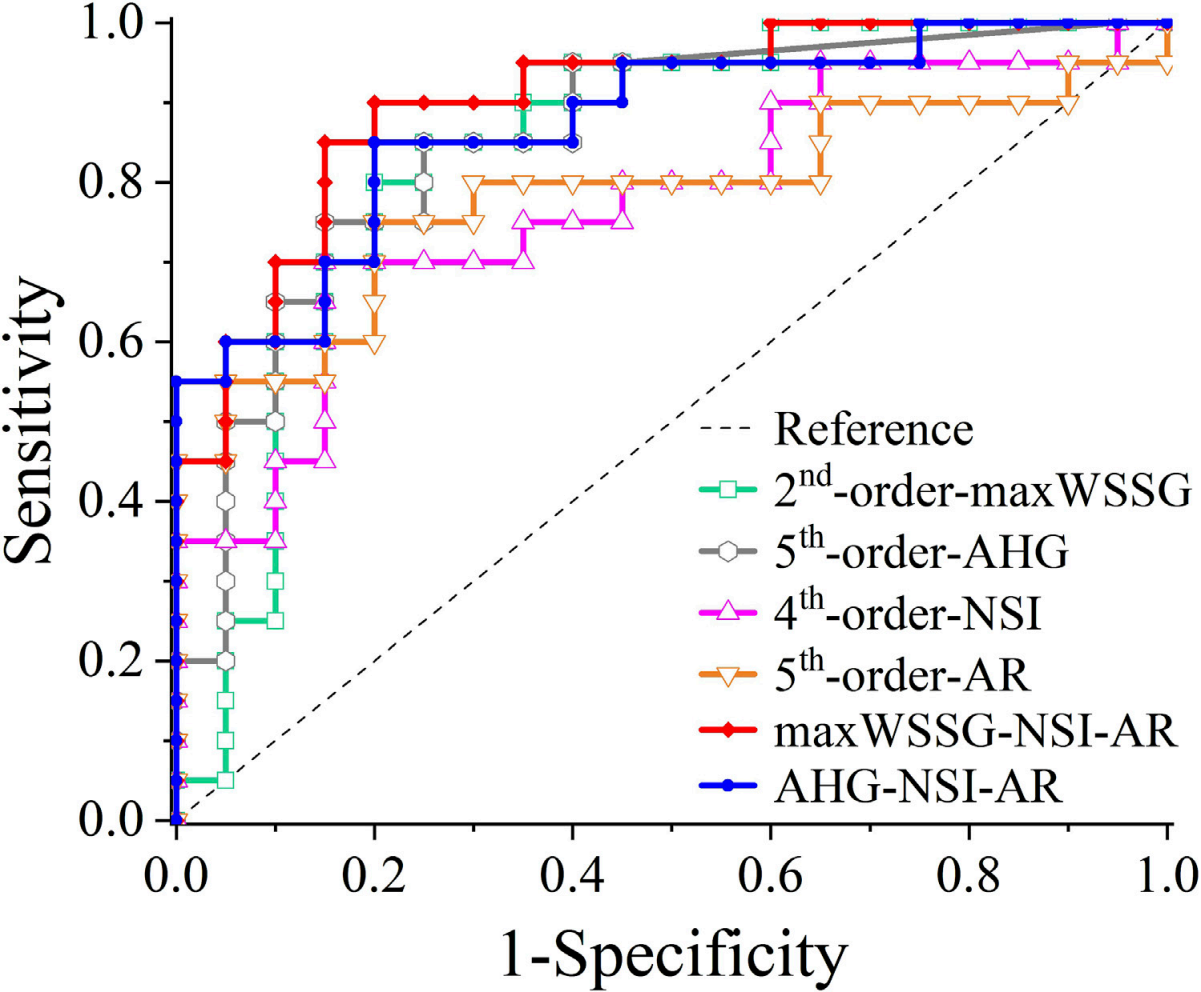
ROC curves of different logistic regression models: maxWSSG, AHG, NSI, AR, maxWSSG-NSI-AR, AHG-NSI-AR.

When the two multivariate regression models were examined with ROC analysis, the values of AUC were 0.9025 and 0.8725, respectively. The detailed results of ROC analysis for the multivariate regression models are listed together with those for the single-variate regression models in Table 5, and the corresponding ROC curves are plotted in Figure 5. It is clear that the multivariate models significantly outperformed any single-variate models, which demonstrates the potential of combining multiple hemodynamic and morphological parameters through multivariate modeling for improving the prediction of aneurysm rupture.

**TABLE 5:** The areas under the ROC curves (AUCs) of different logistic regression models: maxWSSG, AHG, NSI, AR, maxWSSG-NSI-AR, AHG-NSI-AR.

Model	AUC	Threshold	Sensitivity	Specificity
2nd-order-maxWSSG	0.8400	0.427	0.80	0.80
5th-order-AHG	0.8550	0.331	0.85	0.75
4th-order-NSI	0.7725	0.530	0.70	0.85
5th-order-AR	0.7825	0.371	0.75	0.80
maxWSSG-NSI-AR	0.9025	0.337	0.90	0.80
AHG-NSI-AR	0.8725	0.445	0.85	0.80

## 4 Discussion

While numerous studies have been conducted to explore the associations between hemodynamic factors and the rupture status/event of IAs, the outcomes of many studies might have been compromised by improper hemodynamic modeling. The issue could be especially important for IAs with multiple surrounding arteries, such as ACoA aneurysms. Theoretically, the blood flow patterns in an ACoA aneurysm are determined not solely by the aneurysm morphology but affected considerably by the multi-stream inflows and outflows as well. Our numerical tests on two ACoA aneurysms demonstrated that in comparison with global modeling where an ACoA aneurysm is modeled as part of the systemic cerebral artery network, local modeling of an ACoA aneurysm with the physical boundaries confined to its adjacent arteries could not accurately capture intra-aneurysmal hemodynamic characteristics even though the inflow rates were correctly assigned. Inspired by this finding, we carried out hemodynamic simulations for forty ACoA aneurysms (including twenty ruptured and twenty unruptured aneurysms) based on the global modeling method. Although the global modeling method incurred remarkably increased computational cost, the numerical results would better reflect the *in vivo* hemodynamic conditions. Based on the numerical results, we explored hemodynamic parameters (herein mainly WSS metrics) associated closely with the rupture status of ACoA aneurysms, and subsequently combined the identified hemodynamic parameters with morphological parameters to derived models for predicting the rupture status of ACoA aneurysms via multivariate logistic regression analysis.

The maximum/minimum values (i.e., maxTAWSS, minWSSD, and maxWSSG) and area indices (AHS, ALD, AHG, ALDR, and AHGR) of TAWSS, WSSD, and WSSG were found to differ significantly between the ruptured and unruptured ACoA aneurysms. The finding regarding the close association of maxTAWSS with aneurysm rupture is consistent with previous findings (Detmer et al., 2019; Xin et al., 2022). Relatively, WSSD and WSSG were less addressed by previous studies. Our study demonstrated that negative WSSD and high WSSG were associated closely with aneurysm rupture as well. Physically, negative WSSD is detected when WSS vectors converge toward a focal region, which has been proved to enhance the accumulation of atherogenic substances (e.g., LDL) on vascular lumen surface (Mazzi et al., 2022; Tian et al., 2023). High WSSG indicates the presence of large spatial variations in the magnitudes of WSS vectors, which has been found to have the effect of upregulating inflammatory marker expression (Rouleau et al., 2010). Given the negative impact of negative WSSD and high WSSG on mass transfer and vascular function, aneurysm walls exposed to negative WSSD and/or high WSSG may be prone to functional and structural degenerations that might increase the risk of rupture. On the other hand, correlation analyses revealed that most of the WSS metrics identified to be associated with aneurysm rupture were strongly inter-correlated, although they have differential physical meanings, which implies that ruptured ACoA aneurysms might suffer from multiple coexisting patterns of WSS disturbance. Interestingly, low WSS and high OSI were not found to be associated with aneurysm rupture, although they have been widely employed to predict the rupture risk of IAs (Liang et al., 2018), including ACoA aneurysms (Detmer et al., 2019). Reasons underlying the discrepancy between our findings and previous ones remain unknown and may involve multiple factors, such as the location of aneurysm, composition of samples, and methods of hemodynamic modeling, which are usually study-specific. In addition, the relatively small size of ACoA aneurysms at rupture compared to other aneurysms (Forget et al., 2001; Jeong et al., 2009; Joo et al., 2009) may be a contributing factor, since small bifurcation aneurysms provide limited sac space for the development of flow recirculation and separation to generate low and oscillatory WSS.

As for morphological factors, four morphological parameters (i.e., NSI, HWR, AR, and BF) were identified to be associated closely with aneurysm rupture, among which NSI and AR exhibited the strongest association. The results were basically consistent with the findings of previous studies (Liang et al., 2018; Detmer et al., 2019; Xin et al., 2022), suggesting that irregular shape predisposes aneurysms to rupture. An exception was the volume of aneurysm (V_aneu_), which did not exhibit a statistically significant difference between the ruptured and unruptured ACoA aneurysms, although it has been suggested to be a general risk factor for aneurysm rupture (Detmer et al., 2019; Xin et al., 2022). A potential explanation for this disparity may be similar to that for the irrelevance of ACoA aneurysm rupture to low WSS and high OSI. The specific location of ACoA aneurysm and the small size at the moment of rupture determine that the inflow conditions and irregular aneurysm shape (e.g., measured by NSI and AR), rather than the size of aneurysm, dominate the occurrence of aneurysm rupture-promoting flow disturbances.

Most of the identified hemodynamic and morphological parameters could independently discriminate the rupture status of aneurysms with an AUC of > 0.7 in ROC analysis, and the predictive capability of each individual parameter could be further improved (with the AUC increasing up to 0.85) if a nonlinear model was constructed via single-variate logistic regression analysis. Nevertheless, synthesizing multiple independent hemodynamic and morphological parameters in a single model by multivariate logistic regression analysis could yield better outcomes of prediction, e.g., AUC = 0.9025 for the combination of maxWSSG, NSI and AR, and AUC = 0.8725 for the combination of AHG, NSI and AR. These results suggest that hemodynamic and morphological factors are complementary in assessing the rupture risk of ACoA aneurysms. In addition, although the maxWSSG-NSI-AR model had larger AUC, we prefer to recommend the use of the AHG-NSI-AR model because maxWSSG is a local value susceptible to the influence of errors in model reconstruction and boundary condition prescription, whereas, AHG, which reflects the total area of aneurysm walls exposed to high WSSG, is relatively insensitive to such errors.

While the results of the present study provide valuable insights for exploiting the hemodynamic and morphological characteristics to assess the rupture risk of ACoA aneurysms, they must be considered in the context of certain limitations. A major limitation is due to the small data size. Although the forty ACoA aneurysms involved in the study enabled statistical analysis, the number of aneurysms could not support the derivation and testing of higher-order multivariate regression models for predicting rupture events given the requirement on sample size in regression analysis. Ideally, two datasets will be required if larger-scale data would be available, with one dataset for deriving while the other dataset for testing models. From the results of single-variate regression analysis, higher-order models exhibited better performances than the first-order models, in this sense, there exists the potential for further improving the performances of multivariate regression models by including more high-order terms, provided sufficient data would be available. Another limitation is related to the rigid-wall assumption. The walls of cerebral arteries and IAs are compliant in nature, which deform dynamically under pulsatile flow conditions. Previous studies comparing rigid and compliant IA models have demonstrated that rigid models may considerably underestimate near-wall flow velocity oscillation and overestimate TAWSS in some regions (Bazilevs et al., 2010; Torii et al., 2011; Tupin et al., 2020; Sun et al., 2022). Fortunately, the discrepancies between the two types of models were small (usually <10%) in terms of the predicted results of key WSS metrics (e.g., space-averaged TAWSS, maxTAWSS). In consideration of the overall small size of ACoA aneurysms investigated by the present study, the impact of wall deformation on intra-aneurysmal hemodynamic characteristics should be secondary and would not pose substantial influence on the major findings of our study. Nonetheless, accounting for the influence of wall deformation with advanced modeling methods, such as the fluid-structure interaction method, would be expected to further improve the physiological fidelity of numerical results. In addition, we could not identify the specific sites of ruptured aneurysm walls based on CTA images and therefore could not explore the specific hemodynamic features at the rupture sites. This limitation may be addressed if other medical images (e.g., images taken by intracranial photography during surgery (Li et al., 2018) that enable the identification of rupture site were available).

## 5 Conclusion

A computational model-based study has been carried out to investigate hemodynamic differences between ruptured and unruptured ACoA aneurysms, and derive multivariate models for discriminating the rupture status of aneurysms. The study firstly demonstrated the importance of modeling ACoA aneurysm as part of the cerebral artery network in order to accurately capture intra-aneurysmal hemodynamic characteristics, offering a methodological reference for relevant studies in this field. Indices of WSSD and WSSG, rather than traditional WSS metrics like low WSS area and high OSI, were found to differ significantly between the ruptured and unruptured ACoA aneurysms, which implies that ACoA aneurysms, which often rupture at small size, may differ from aneurysms in other cerebral arteries with respect to flow disturbances driving aneurysm wall weakening and rupture. In addition, multivariate regression models composed of polynomials of hemodynamic and morphological parameters were proved to outperform single-variate models in discriminating the rupture status of ACoA aneurysms, suggesting that the rupture risk of ACoA aneurysms should be assessed on the basis of a synthetical consideration of hemodynamic and morphological factors. On the other hand, it should be stressed that the derivation of multivariate regression models was limited by the small data size (herein n = 40), which could be further improved if larger-scale data were available.

## Data Availability

The data analyzed in this study is subject to the following licenses/restrictions: The data that support the findings of this study are available from the corresponding author upon reasonable request. Requests to access these datasets should be directed to fuyouliang@sjtu.edu.cn.

## References

[B1] Alastruey J. Moore S. M. Parker K. H. David T. Peiró J. Sherwin S. J. (2008). Reduced modelling of blood flow in the cerebral circulation: coupling 1-D, 0-D and cerebral auto-regulation models. Int. J. Numer. Methods Fluids 56, 1061–1067. 10.1002/fld.1606

[B2] Alastruey J. Parker K. H. Peiro J. Byrd S. M. Sherwin S. J. (2007). Modelling the circle of Willis to assess the effects of anatomical variations and occlusions on cerebral flows. J. Biomechanics 40, 1794–1805. 10.1016/j.jbiomech.2006.07.008 17045276

[B3] Amenta P. S. Yadla S. Campbell P. G. Maltenfort M. G. Dey S. Ghosh S. (2012). Analysis of nonmodifiable risk factors for intracranial aneurysm rupture in a large, retrospective cohort. Neurosurgery 70, 693–701. 10.1227/neu.0b013e3182354d68 21904261

[B4] Babyak M. A. (2004). What you see may not be what you get: a brief, nontechnical introduction to overfitting in regression-type models. Psychosom. Med. 66, 411–421. 10.1097/01.psy.0000127692.23278.a9 15184705

[B5] Backes D. Vergouwen M. D. I. Velthuis B. K. van der Schaaf I. C. Bor A. S. E. Algra A. (2014). Difference in aneurysm characteristics between ruptured and unruptured aneurysms in patients with multiple intracranial aneurysms. Stroke 45, 1299–1303. 10.1161/strokeaha.113.004421 24652309

[B6] Bazilevs Y. Hsu M.-C. Zhang Y. Wang W. Liang X. Kvamsdal T. (2010). A fully-coupled fluid-structure interaction simulation of cerebral aneurysms. Comput. Mech. 46, 3–16. 10.1007/s00466-009-0421-4

[B7] Brisman J. L. Song J. K. Newell D. W. (2006). Cerebral aneurysms. N. Engl. J. Med. 355, 928–939. 10.1056/nejmra052760 16943405

[B8] Castro M. Putman C. M. Cebral J. (2006). Computational fluid dynamics modeling of intracranial aneurysms: effects of parent artery segmentation on intra-aneurysmal hemodynamics. Am. J. Neuroradiol. 27, 1703–1709. 16971618 PMC8139802

[B9] Cebral J. R. Mut F. Weir J. Putman C. M. (2011). Association of hemodynamic characteristics and cerebral aneurysm rupture. Am. J. Neuroradiol. 32, 264–270. 10.3174/ajnr.a2274 21051508 PMC3070915

[B10] Chen J. Li M. Zhu X. Chen Y. Zhang C. Shi W. (2020). Anterior communicating artery aneurysms: anatomical considerations and microsurgical strategies. Front. Neurology 11, 1020. 10.3389/fneur.2020.01020 PMC7509403 33013671

[B11] Chien A. Tateshima S. Castro M. Sayre J. Cebral J. ViñUELA F. (2008). Patient-specific flow analysis of brain aneurysms at a single location: comparison of hemodynamic characteristics in small aneurysms. Med. Biol. Eng. Comput. 46, 1113–1120. 10.1007/s11517-008-0400-5 18931868

[B12] Chien A. Xu M. Yokota H. Scalzo F. Morimoto E. Salamon N. (2018). Nonsphericity index and size ratio identify morphologic differences between growing and stable aneurysms in a longitudinal study of 93 cases. Am. J. Neuroradiol. 39, 500–506. 10.3174/ajnr.a5531 29371255 PMC7655307

[B13] Detmer F. J. Chung B. J. Jimenez C. Hamzei-Sichani F. Kallmes D. Putman C. (2019). Associations of hemodynamics, morphology, and patient characteristics with aneurysm rupture stratified by aneurysm location. Neuroradiology 61, 275–284. 10.1007/s00234-018-2135-9 30456458 PMC6403015

[B14] Forget T. R. JR. Benitez R. Veznedaroglu E. Sharan A. Mitchell W. Silva M. (2001). A review of size and location of ruptured intracranial aneurysms. Neurosurgery 49, 1322–1326. 10.1097/00006123-200112000-00006 11846931

[B15] Hassan T. Hassan A. A. Ahmed Y. M. (2011). Influence of parent vessel dominancy on fluid dynamics of anterior communicating artery aneurysms. Acta Neurochir. 153, 305–310. 10.1007/s00701-010-0824-1 20924768

[B16] Hodis S. Kargar S. Kallmes D. F. Dragomir-Daescu D. (2015). Artery length sensitivity in patient-specific cerebral aneurysm simulations. Am. J. Neuroradiol. 36, 737–743. 10.3174/ajnr.a4179 25500310 PMC7964310

[B17] Hua Y. Oh J. H. Kim Y. B. (2015). Influence of parent artery segmentation and boundary conditions on hemodynamic characteristics of intracranial aneurysms. Yonsei Med. J. 56, 1328. 10.3349/ymj.2015.56.5.1328 26256976 PMC4541663

[B18] Jenkins D. G. Quintana-Ascencio P. F. (2020). A solution to minimum sample size for regressions. PLOS ONE 15, e0229345. 10.1371/journal.pone.0229345 32084211 PMC7034864

[B19] Jeong Y.-G. Jung Y.-T. Kim M.-S. Eun C.-K. Jang S.-H. (2009). Size and location of ruptured intracranial aneurysms. J. Korean Neurosurg. Soc. 45, 11. 10.3340/jkns.2009.45.1.11 19242565 PMC2640825

[B20] Johnston B. M. Johnston P. R. Corney S. Kilpatrick D. (2004). Non-Newtonian blood flow in human right coronary arteries: steady state simulations. J. Biomechanics 37, 709–720. 10.1016/j.jbiomech.2003.09.016 15047000

[B21] Joo S. W. Lee S.-I. Noh S. J. Jeong Y. G. Kim M. S. Jeong Y. T. (2009). What is the significance of a large number of ruptured aneurysms smaller than 7 mm in diameter? J. Korean Neurosurg. Soc. 45, 85. 10.3340/jkns.2009.45.2.85 19274117 PMC2651552

[B22] Karmonik C. Yen C. Grossman R. G. Klucznik R. Benndorf G. (2009). Intra-aneurysmal flow patterns and wall shear stresses calculated with computational flow dynamics in an anterior communicating artery aneurysm depend on knowledge of patient-specific inflow rates. Acta Neurochir. 151, 479–485. 10.1007/s00701-009-0247-z 19343271

[B23] Ku D. N. Giddens D. P. Zarins C. K. Glagov S. (1985). Pulsatile flow and atherosclerosis in the human carotid bifurcation. Positive correlation between plaque location and low oscillating shear stress. Arteriosclerosis Official J. Am. Heart Assoc. Inc. 5, 293–302. 10.1161/01.atv.5.3.293 3994585

[B24] Kwak R. Niizuma H. Suzuki J. (1980). Hemodynamics in the anterior part of the circle of willis in patients with intracranial aneurysms: a study by cerebral angiography. Tohoku J. Exp. Med. 132, 69–73. 10.1620/tjem.132.69 7209970

[B25] Liang F. Fukasaku K. Liu H. Takagi S. (2011). A computational model study of the influence of the anatomy of the circle of willis on cerebral hyperperfusion following carotid artery surgery. Biomed. Eng. OnLine 10, 84. 10.1186/1475-925x-10-84 21943370 PMC3203260

[B26] Liang F. Liu X. Yamaguchi R. Liu H. (2016). Sensitivity of flow patterns in aneurysms on the anterior communicating artery to anatomic variations of the cerebral arterial network. J. biomechanics 49, 3731–3740. 10.1016/j.jbiomech.2016.09.031 27743630

[B27] Liang L. Steinman D. A. Brina O. Chnafa C. Cancelliere N. M. Pereira V. M. (2018). Towards the Clinical utility of CFD for assessment of intracranial aneurysm rupture–a systematic review and novel parameter-ranking tool. J. neurointerventional Surg. 11, 153–158. 10.1136/neurintsurg-2018-014246 30341160

[B28] Li J. Wang S. Lu G. Zhang X. (2014). Hemodynamics modeling and simulation of anterior communicating artery aneurysms. Adv. Mech. Eng. 6, 908357. 10.1155/2014/908357

[B29] Li M. Wang J. Liu J. Zhao C. Yang X. (2018). Hemodynamics in ruptured intracranial aneurysms with known rupture points. World Neurosurg. 118, e721–e726. 10.1016/j.wneu.2018.07.026 30010065

[B30] Lu H.-T. Tan H.-Q. Gu B.-X. Wu W. Li M.-H. (2013). Risk factors for multiple intracranial aneurysms rupture: a retrospective study. Clin. Neurology Neurosurg. 115, 690–694. 10.1016/j.clineuro.2012.08.011 22921040

[B31] Ma B. Harbaugh R. E. Raghavan M. L. (2004). Three-dimensional geometrical characterization of cerebral aneurysms. Ann. Biomed. Eng. 32, 264–273. 10.1023/b:abme.0000012746.31343.92 15008374

[B32] Malek A. M. Alper S. L. Izumo S. (1999). Hemodynamic shear stress and its role in atherosclerosis. J. Am. Med. Assoc. 282, 2035–2042. 10.1001/jama.282.21.2035 10591386

[B33] Marc A. L. Bichun O. Michael C. (2012). The role of circle of Willis anomalies in cerebral aneurysm rupture. J. NeuroInterventional Surg. 4, 22–26. 10.1136/jnis.2010.004358 21990452

[B34] Mazzi V. de Nisco G. Calò K. Chiastra C. Daemen J. Steinman D. A. (2022). Divergence of the normalized wall shear stress as an effective computational template of low-density lipoprotein polarization at the arterial blood-vessel wall interface. Comput. Methods Programs Biomed. 226, 107174. 10.1016/j.cmpb.2022.107174 36223707

[B35] Mehdi N. Nicole M. C. Olivier B. Pierre B. Maria I. V. Benedicte M. A. D. (2021). How patient-specific do internal carotid artery inflow rates need to be for computational fluid dynamics of cerebral aneurysms? J. NeuroInterventional Surg. 13, 459–464. 10.1136/neurintsurg-2020-015993 32732256

[B36] Metaxa E. Tremmel M. Natarajan S. K. Xiang J. Paluch R. A. Mandelbaum M. (2010). Characterization of critical hemodynamics contributing to aneurysmal remodeling at the basilar terminus in a rabbit model. Stroke 41, 1774–1782. 10.1161/strokeaha.110.585992 20595660 PMC2913882

[B37] Morita A. Kirino T. Hashi K. Aoki N. Fukuhara S. Hashimoto N. (2012). The natural course of unruptured cerebral aneurysms in a Japanese cohort. N. Engl. J. Med. 366, 2474–2482. 10.1056/nejmoa1113260 22738097

[B38] Myers J. Moore J. Ojha M. Johnston K. Ethier C. (2001). Factors influencing blood flow patterns in the human right coronary artery. Ann. Biomed. Eng. 29, 109–120. 10.1114/1.1349703 11284665

[B39] Nagargoje M. S. Mishra D. K. Gupta R. (2021). Pulsatile flow dynamics in symmetric and asymmetric bifurcating vessels. Phys. Fluids 33, 071904. 10.1063/5.0056414

[B40] Niederberger E. Gauvrit J. Y. Morandi X. Carsin-Nicol B. Gauthier T. Ferré J. C. (2010). Anatomic variants of the anterior part of the cerebral arterial circle at multidetector computed tomography angiography. J. Neuroradiol. 37, 139–147. 10.1016/j.neurad.2009.12.004 20346510

[B41] Pahlevan N. M. Amlani F. Hossein Gorji M. Hussain F. Gharib M. (2011). A physiologically relevant, simple outflow boundary model for truncated vasculature. Ann. Biomed. Eng. 39, 1470–1481. 10.1007/s10439-011-0246-0 21240638

[B42] Park Y. K. Yi H.-J. Choi K.-S. Lee Y.-J. Chun H.-J. (2018). Intraprocedural rupture during endovascular treatment of intracranial aneurysm: clinical results and literature review. World Neurosurg. 114, e605–e615. 10.1016/j.wneu.2018.03.040 29548958

[B43] Peduzzi P. Concato J. Kemper E. Holford T. R. Feinstein A. R. (1996). A simulation study of the number of events per variable in logistic regression analysis. J. Clin. Epidemiol. 49, 1373–1379. 10.1016/s0895-4356(96)00236-3 8970487

[B44] Pereira V. M. Brina O. Marcos Gonzales A. Narata A. P. Bijlenga P. Schaller K. (2013). Evaluation of the influence of inlet boundary conditions on computational fluid dynamics for intracranial aneurysms: a virtual experiment. J. Biomechanics 46, 1531–1539. 10.1016/j.jbiomech.2013.03.024 23602597

[B45] Reymond P. Merenda F. Perren F. RüFENACHT D. Stergiopulos N. (2009). Validation of a one-dimensional model of the systemic arterial tree. Am. J. Physiology-Heart Circulatory Physiology 297, H208–H222. 10.1152/ajpheart.00037.2009 19429832

[B46] Rouleau L. Rossi J. Leask R. L. (2010). The response of human aortic endothelial cells in a stenotic hemodynamic environment: effect of duration, magnitude, and spatial gradients in wall shear stress. J. Biomechanical Eng. 132, 071015. 10.1115/1.4001217 20590293

[B47] SchröDER F. Regelsberger J. Westphal M. Freckmann N. Grzyska U. Herrmann H. (1997). Asymptomatic cerebral aneurysms--surgical and endovascular therapy options. Wien. Med. Wochenschr. 147, 159–162. 9297364

[B48] Shimogonya Y. Ishikawa T. Imai Y. Matsuki N. Yamaguchi T. (2009). Can temporal fluctuation in spatial wall shear stress gradient initiate a cerebral aneurysm? A proposed novel hemodynamic index, the gradient oscillatory number (GON). J. Biomechanics 42, 550–554. 10.1016/j.jbiomech.2008.10.006 19195658

[B49] Starke R. M. Chalouhi N. Ali M. S. Jabbour P. M. Tjoumakaris S. I. Gonzalez L. F. (2013). The role of oxidative stress in cerebral aneurysm formation and rupture. Curr. Neurovascular Res. 10, 247–255. 10.2174/15672026113109990003 PMC3845363 23713738

[B50] Steinman D. A. Pereira V. M. (2019). How patient specific are patient-specific computational models of cerebral aneurysms? An overview of sources of error and variability. Neurosurg. Focus 47, E14. 10.3171/2019.4.focus19123 31261118

[B51] Stergiopulos N. Young D. Rogge T. (1992). Computer simulation of arterial flow with applications to arterial and aortic stenoses. J. biomechanics 25, 1477–1488. 10.1016/0021-9290(92)90060-e 1491023

[B52] Stojanović N. N. Kostić A. Mitić R. Berilažić L. Radisavljević M. (2019). Association between circle of willis configuration and rupture of cerebral aneurysms. Medicina 55, 338. 10.3390/medicina55070338 31277348 PMC6681035

[B53] Sun H. T. Sze K. Y. Chow K. W. On Tsang A. C. (2022). A comparative study on computational fluid dynamic, fluid-structure interaction and static structural analyses of cerebral aneurysm. Eng. Appl. Comput. Fluid Mech. 16, 262–278. 10.1080/19942060.2021.2013322

[B54] Tarulli E. Sneade M. Clarke A. Molyneux A. J. Fox A. J. (2014). Effects of circle of willis anatomic variations on angiographic and clinical outcomes of coiled anterior communicating artery aneurysms. Am. J. Neuroradiol. 35, 1551–1555. 10.3174/ajnr.a3991 24948501 PMC7964461

[B55] Tian Y. Li X. Zhao B. Zhang J. Liang F. (2023). Influence of morphological characteristics on the deposition of low-density lipoprotein in intracranial bifurcation aneurysms. Phys. Fluids 35, 081905. 10.1063/5.0159985

[B56] Torii R. Oshima M. Kobayashi T. Takagi K. Tezduyar T. E. (2011). Influencing factors in image‐based fluid–structure interaction computation of cerebral aneurysms. Int. J. Numer. Methods Fluids 65, 324–340. 10.1002/fld.2448

[B57] Tupin S. Saqr K. M. Ohta M. (2020). Effects of wall compliance on multiharmonic pulsatile flow in idealized cerebral aneurysm models: comparative PIV experiments. Exp. Fluids 61, 164. 10.1007/s00348-020-02998-4

[B58] van Gijn J. Kerr R. S. Rinkel G. J. (2007). Subarachnoid haemorrhage. Lancet 369, 306–318. 10.1016/s0140-6736(07)60153-6 17258671

[B59] Vittinghoff E. Mcculloch C. E. (2007). Relaxing the rule of ten events per variable in logistic and Cox regression. Am. J. Epidemiol. 165, 710–718. 10.1093/aje/kwk052 17182981

[B60] Xiang J. Natarajan S. K. Tremmel M. Ma D. Mocco J. Hopkins L. N. (2011). Hemodynamic-morphologic discriminants for intracranial aneurysm rupture. Stroke 42, 144–152. 10.1161/strokeaha.110.592923 21106956 PMC3021316

[B61] Xin S. Chen Y. Zhao B. Liang F. (2022). Combination of morphological and hemodynamic parameters for assessing the rupture risk of intracranial aneurysms: a retrospective study on mirror middle cerebral artery aneurysms. J. Biomechanical Eng. 144, 081006. 10.1115/1.4053793 35147191

[B62] Xu L. Liang F. Gu L. Liu H. (2018a). Flow instability detected in ruptured versus unruptured cerebral aneurysms at the internal carotid artery. J. biomechanics 72, 187–199. 10.1016/j.jbiomech.2018.03.014 29602477

[B63] Xu L. Liang F. Zhao B. Wan J. Liu H. (2018b). Influence of aging-induced flow waveform variation on hemodynamics in aneurysms present at the internal carotid artery: a computational model-based study. Comput. Biol. Med. 101, 51–60. 10.1016/j.compbiomed.2018.08.004 30099239

[B64] Yang H. Cho K.-C. Hong I. Kim Y. Kim Y. B. Kim J.-J. (2024). Influence of circle of Willis modeling on hemodynamic parameters in anterior communicating artery aneurysms and recommendations for model selection. Sci. Rep. 14, 8476. 10.1038/s41598-024-59042-2 38605063 PMC11009257

[B65] Zhou X. Yin L. Xu L. Liang F. (2020). Non-periodicity of blood flow and its influence on wall shear stress in the carotid artery bifurcation: an *in vivo* measurement-based computational study. J. Biomechanics 101, 109617. 10.1016/j.jbiomech.2020.109617 31959390

